# Natural Killer Cells Expanded and Preactivated Exhibit Enhanced Antitumor Activity against Different Tumor Cells in Vitro

**DOI:** 10.31557/APJCP.2020.21.6.1595

**Published:** 2020-06

**Authors:** Biplob Kumar Biswas, Sameer Ahmad Guru, Mamta Pervin Sumi, Elvia Jamatia, Rohit Kumar Gupta, Pramod Lali, Bidhan Chandra Konar, Alpana Saxena, Rashid Mir

**Affiliations:** 1 *Department of Biochemistry, Maulana Azad Medical College (MAMC) and Associated Hospitals, New Delhi, India. *; 2 *Multidisciplinary Research Unit (MRU), Maulana Azad Medical College and Associated Hospitals, Bahadur Shah Zafar Marg, New Delhi, 110002, India. *; 3 *Department of Gastroinstestinal Surgery G B Pant Postgraduate Institute of Medical Education and Research (GIPMER), New Delhi, India. *; 4 *Department of Medical Lab Technology, Faculty of Applied Medical Sciences, Prince Fahd Bin Sultan Research chair, University of Tabuk, Saudi Arabia. *

**Keywords:** Natural killer (NK), CD34+ cells, K562 (Chronic Myeloid Leukaemia), T47D, Umbilical Cord Blood (UCB)

## Abstract

One of the emerging treatment strategies for cancer particularly for haematological malignancies is natural killer (NK) cell therapy. However, the availability of a best approach to maximize NK cell anticancer potential is still awaited. It is well established that cytokine-induced memory-like NK cells have the potential to differentiate after a short period of preactivation with interleukins-IL-12, IL-15, and IL-18 and exhibit increased responses to cytokine or activating receptor restimulation for weeks to months after preactivation. We demonstrated that NK cells differentiated from CD34+ cells isolated from cord blood show increased antitumor potential in vitro against different cancer cells. Using flow cytometry, we found that NK cells were able to induce apoptosis in cancer cells in vitro. We further analysed surviving gene expression by quantitative real time PCR and reported that NK cells cause down regulation of survivin gene expression in tumor cells. Therefore, NK cell therapy represents a promising immunotherapy for cancers like AML and other haematological malignancies. It concluded that NK cells can be differentiated from CD34+ cells isolated from cord blood ,are able to induce apoptosis and induce increased antitumor potential in vitro against different cancer cells besides cause downregulation of survivin gene expression in tumor cells. Therefore, NK cell therapy represents a promising immunotherapy for different cancer types and haematological malignancies. Furthers studies are necessary to confirm our findings.

## Introduction

Natural killer (NK) cells are large granular lymphocytes playing role in our defense against certain virus-infected and malignant cells. Natural killer (NK) cells also lyse target cells via antibody-dependent cellular cytotoxicity, a critical mode of action of several therapeutic antibodies used to treat cancer (Rezvani and Rouce, 2015). Natural killer (NK) cells play a critical role in innate immune responses against infected cells and transformed cells (Paul and Lal, 2017) .Many researches highlight on the role of NK cells in hematologic malignancies, particularly in acute leukaemia.NK cells are capable of producing cytokines such as interferon-γ (IFN-γ) and tumour necrosis factor-α (TNF-α) in response to stimuli (Kronstad et al., 2018; Cerwenka and Lanier, 2016). NK cells are considered a part of lymphocytes that account for approximately 10% of blood lymphocytes. NK cells are characterized by expression of CD56 surface antigen and a lack of CD3 antigen. Based on the density of CD56 expression, human NK cells are phenotypically divided into two groups: CD56bright and CD56dim.Of these NK cell populations, CD56dim NK cells comprise up to 90% of NK cells in human peripheral blood mononuclear cells (PBMCs) and are considered the most cytotoxic subset, while CD 56bright NK cells comprise approximately 10% of NK cells in PBMCs and are known as the cytokine-producing subset. NK cells play important role of the first line of defense to infected cells and transformed cells without prior sensitization (Hammer et al., 2018). CD56dimCD16+ NK cells (CD56dim NK cells) are cytotoxic NK cells which are able to cause direct target cell killing through exocytosis of granules containing granzyme B and perforin, activation of TRAIL or FAS/FAS-L cell death pathways or antibody dependent cellular cytotoxicity. CD56 bright CD16-/low NK cells (CD56bright NK cells) which are main cytokine-producing NK cells. Almost 90% of peripheral blood NK cells are CD56dim while CD56bright NK cells mainly reside in lymph nodes (Cooper et al., 2001). The functions of NK cells are regulated by activating and inhibitory receptor signals. In contrast to T cells, NK cells are ready to go and are able to eliminate target cells without prior stimulation. However, they come with enhanced functions only after activation by cytokines, in particular enhanced cytolytic activity and proliferation. NK cells are established potential candidates used for immunotherapy of cancer and their flexibility makes them attractive cells to explore. It has been reported that autologous NK cell therapies are feasible and safe without adverse effects in patients with non-Hodgkin’s lymphoma and breast cancer (Burns et al., 2003). NK cell alloreactivity potential impact in haematopoietic stem cell transplantation (HSCT) was suggested by Valiante and Parham (1997). The proof that allogenic NK cells anti-leukemic activity and impact on the outcome of haploidentical transplantation comes from the studies of Ruggeri et al., (2002). 

The field of NK cell based therapy has recently gained much interest and people are exploring the potential roles that NK cells are capable of playing in the treatment of various diseases (Welsh, 1978). Therefore, NK cell biology has expanded well beyond simply describing the cytotoxic functions of these cells, with new roles attributed to the collection of NK cell produced cytokines and the potential targets NK cells are able to recognize and bind (Cumulated Index Medicus, 1977) NK cells have been known to play important roles in not only viral and tumor resistance but also bacterial and fungal immune responses (Chalifour et al., 2004; Bouzani et al., 2011).NK cells have also been reported to be important in bone marrow rejections and cell engraftment as well (Li et al., 2013; Sun et al., 2012). A number of studies have reported that a variety cancer cell types including that of acute myeloid leukaemia, Hodgkin lymphoma, breast cancer, refractory lymphoma, ovarian and renal cell carncinoma can be targeted by allogeneic NK cells (Cumulated Index Medicus, 1992; Bachanova et al., 2010). Some studies have also shown the importance of NK cell expansion in vivo accomplished by infusion of interleukin-2 (IL-2) (Bachanova et al., 2014). NK cell therapy has also been reported to be of interest in treating glioma and neuroblastoma (Ishikawa et al., 2004; Tarek et al., 2012). Given the importance of NK cells as anti-cancer entities, a method of therapy that shall permit for the delivery of a large number of tumours naive activated NK cells to cancer patients and the ability to overcome the immunosuppressive tumour environment would have great potential (Gillgrass et al., 2011).

A sufficient amount of NK cells that too with a high cytotoxicity is required for conducting clinical applications about NK cell-based immunotherapy. Umbilical cord blood (UCB), bone marrow, peripheral blood (PB) and embryonic stem cells are the different sources for obtaining NK cells. Of these sources, UCB not only provides higher proportion of NK cells but is also an easy source of NK cell. Moreover, UCB can be stored and preserved for a long time to be used as readily source of NK-cell based therapy. Nevertheless, although UCB is having greater amount of NK cells in contrast to PB, the number of NK cells actually obtained is really small because of the limited volume of UCB which presents a major hindrances in providing adequate amount of NK cells for clinical applications.

It was elucidated in our study that the NK cells can be differentiated from CD34+ cells isolated from cord blood and is capable to induce apoptosis with increased antitumor potential in vitro against different cancer cells via downregulation of survivin gene expression in tumor cells. Hence, following cytokine stimulation, the cytotoxicity of UCB NK cells can be rapidly increased to levels that are comparable to PB NK cells. Therefore, NK cell therapy represents a promising immunotherapy for different cancer types and haematological malignancies. Further studies are necessary to confirm our findings. Therefore, the aim of this study was to generate NK cells by invitro differentiation of human derived UCB CD34 cells and to expand these NK cells in vitro using the best available methodology and finally test the cytotoxic potential of these NK cells against different cancer cell lines.

## Materials and Methods

*Methodology*

This study was conducted after the approval by the institutional ethics committee (IEC), MAMC and associated hospitals.This study was performed at the Department of Biochemistry in collaboration with Departments of Gynaecology and Pathology, Maulana Azad Medical College (MAMC) and Associated Hospitals, New Delhi, India 

*Cell lines utilized*

Several cell lines were used in this study including –K562 (Chronic Myeloid Leukaemia), T47D (Human Breast Adenocarcinoma) -established from the pleural effusion of a ductal carcinoma of the breast of a 54-year-old female. The cells carry receptors for a variety of steroids.U87MG (Glioblastoma) and T47D (Human Breast Adenocarcinoma) used in this study were obtained from National Centre Cell for cell Science (NCCS), Pune, India. K562 and T-47D were cultured in Rosewell Park Memorial Institute (RPMI 1640) while U87 was cultured in Dulbecco’s Modified Eagle’s Medium (DMEM) media containing 10% Fetal Bovine Serum, 100 U/mL Penicillin, 100 µg/mL Streptomycin, 250 ng/mL Amphotericin, 250 µg/mL Gentamycin and 2mM L-glutamine. Cell lines were maintained at 37^o^C in a humid atmosphere containing 5% CO_2_. On attaining 70-90% confluence, subculturing was performed after cell counting and viability testing.

*Umbilical Cord Blood Collection*

Umbilical Cord Blood (UCB) units were obtained at birth on normal full term delivery after written informed consent regarding scientific use from Department of Gynaecology, Lok Nayak Jay Prakash (LNJP) Hospital (New Delhi, India). The use of these Umbilical Cord Blood (UCB) units for this research was approved by Institutional Ethics Review Board, Maulana Azad Medical College and Associated LNJP Hospital. After collection, the UCB samples were stored at room temperature and processed within 24 hours.

*Isolation of mononuclear cells from cord blood*

Once collected, the Umbilical Cord Blood (UCB) was separated out over a Ficoll Hypaque density gradient (Sigma). Ficoll Hypaque use is facilitated by Ficoll-Hypaque density gradient centrifugation-a simple and rapid method of purifying peripheral blood mononuclear cells (PBMC) that takes advantage of the density differences between mononuclear cells and other elements found in the blood sample. Thus, cells are distributed in the solution in layers based on the differences in their density/size. After centrifugation at 400g for 40 minutes at 20^o^C, the interface mononuclear cells were collected in separate tube, washed twice and were resuspended in Iscove’s Modified Dulbecco’s Media (IMDM) containing 20% heat inactivated foetal calf serum. The cell viability was assessed by trypan blue exclusion test. The Trypan Blue dye exclusion test is used to determine the number of viable cells present in a cell suspension.

CD34+ CELLS COUNT : The presence of CD34+ cells was determined by microscopic immunoflorescence and flow cytometry (FCM). 

Microscopic immunoflorescence: The CD34+ antigen is very highly expressed in pluripotent stem cells and its expression decreases gradually towards terminal differentiation into more mature cells. For immunoflorescence, monolayer of cell smear, without losing their morphology, was prepared using an auto slide smear system called cytocentrifuge. The cytocentrifuge is designed for concentration of biological samples on a visible surface for the microscope and its subsequent identification and characterisation .This reliable bench top centrifuge provides economical thin-layer preparations from any liquid matrix especially hypocellular fluid such as cell suspension. To prepare cell smear, the mononuclear cells were counted and viability assessed using Trypan blue exclusion assay. The cell concentration was 1,500 cells/µl and we used 400 µl cell suspensions for cyto-centrifugation. The contents were centrifuged at 2,000 rpm for 10 minutes. Next, the cells were fixed using 10% formaldehyde, prepared in PBS, for 10 minutes at room temperature. Blocking with 10% FBS prepared in PBS was performed for 20 minutes at 37^o^C to minimize nonspecific adherence of antibodies and preventing generation of false positive signals. The blocking buffer was washed with 1X PBS before staining step. Finally, the smear was stained with conjugated primary antibodies for 1hr. The slides were then washed three times for five minutes with 1X PBS and after drying the slides were visualised under florescent microscope.

*Flow cytometric analysis for expression of CD56+CD3- NK cells*

NK T cells are a heterogeneous T cell population characterized by the co-expression of αβ or γδ TCRs and various NK receptors, including CD16, CD56, CD161, CD94, CD158a and CD158b . Human NK cells express CD16 and CD56 which are (massively) being used as a major hallmark for the NK cell. After performing cell counts and assessing cell viability, 100 µl of cell sample suspension (density 1x10^2^ cells/µl) was added to the fluorochrome-conjugated monoclonal antibodies; CD34-PE, anti-HLA-DR-FITC, CD45-ECD, CD3-PC5 and CD56-PC7 (Beckman Coulter, Inc).The antibodies were diluted in accordance with the manufacturer’s specification. The samples were centrifuged at 1,500 rpm for 5 minutes after a dark incubation for 20 minutes. The suspension was removed after centrifugation and re-suspended with 1 ml of isotone or sheath fluid for washing. After complete wash, the samples were put into Beckman Coulter Flow analyzer, the data acquired and analyses were performed.

*Expansion of CD34+ progenitor cells and NK cell differentiation from cord blood mononuclear cells*

a) Expansion of CD34+ UCB mononuclear cells:

Umbilical Cord Blood (UCB) mononuclear cells (density 1x10^5^ cells/ml) were plated into 25 cm^2^ culture flasks in the Stemline IITM stem cell expansion medium containing 20 mg/mL ascorbic acid, 50 µmol/L ethanolamine, 50 µmol/L sodium selenite, 25 µmol/L β-mercapto-ethanol, 100U/mL penicillin, 100µg/L streptomycin and 2mmol/L L-Glutamine. For each experiment, a 10ml of expansion medium was warmed to 37^o^C. Five ml of the medium was pipette out in 25 cm^2^ culture plates to which 10% foetal bovin serum and a low dose cytokine cocktail consisting of 10 pg/ml GM-CSF , 250 pg/ml G-CSF, 50 pg/ml LIF, 200 pg/ml MIP-1α and 50 pg/ml IL-6 was added. Cell culture was refreshed with new medium every 2-3 days and plates were maintained at 37°C in an atmosphere containing 5% CO_2_ for 10 days. Following the incubation period, the expanded total nucleated cells were counted by using trypan blue assay and the differentiation step was monitored weekly, analyzed for cell numbers and cell surface marker CD34, HLA-DR, CD56, CD3 and CD56 using FCM.

*b) Differentiation of expanded cells into NK cell*

Expanded umbilical cord blood mononuclear cells were differentiated and further expanded using NK cell differentiation medium which consisted of same basal medium as used for expansion supplemented with 10% FBS, the low dose cytokine cocktail as previously mentioned and a new high dose cytokine cocktail which consisted of 20 ng/mL IL-7, 20 ng/mL SCF, 1,000 U/mL IL-2 and 20 ng/mL IL-15. The medium was refreshed twice a week from day 10 onwards and plates were maintained at 37^o^C in an atmosphere containing 5% CO_2_ for 18 days. Cell number, expression of cell surface markers and purity was determined by FCM.

*Cell proliferation assays*

Tumor cells (T47D, U87 and K562) were seeded at equal density (1×10^5 ^cells/ml) in 96 well plates with appropriate medium and incubated at 37^o^C overnight prior to NK cell treatment. Next these tumor cells were treated with different density of NK cells (1x10^4^, 2x10^4^, 4x10^4^ cells/ml) and were further incubated for 48hrs. All the treatments were performed in triplicates and repeated three times. Mean ± SD for each NK cell treatment was determined. The cell viability was determined using MTT assay and apoptosis was measured with Annexin V-FITC assay.

*MTT assay*

The MTT assay is used to measure cellular metabolic activity as an indicator of cell viability, proliferation and cytotoxicity. Tetrazolium dye assays can also be used to measure cytotoxicity (loss of viable cells) or cytostatic activity (shift from proliferation to quiescence) of potential medicinal agents and toxic materials. MTT assays are usually done in the dark since the MTT reagent is sensitive to light. The colorimetric MTT assay based on selective ability of living cells to reduce the yellow salt MTT [3-(4,5-dimethylethiazol-2,5-diphenyltetrazolium bromide] to formazan, was used as a cytotoxicity assay according to the standardized protocol. Briefly, after incubation period of tumor cell treatment with different NK cell density 10µL of MTT (Sigma M2128, 5 mg/ml) was added to each well and incubated for 2hrs at 37^o^C and 5% CO_2_. After two hour incubation, the fomazan crystals were dissolved in 150µL/well dimehtylsulphoxide (100%; Sigma D 8779 ACS). The absorbance was recorded on microplate spectrophotometer (Titertek Multiscan MCC 340, Flow Laboratory) at a wave length of 570 nm. Cell viability (in percentage, %) was depicted as ratio of absorbance (A570nm) in treated cells to absorbance in control cells (DMSO) (A570 nm). 

Cell viability (%) = A570nm (Sample)/ A570nm (Control DMSO) X 100

Flow cytometry measurement of apoptosis (Annexin V-FITC assay):

Equal density (1×10^5^cells/ml) of tumor cells treated with different NK cell density as described above. After designated time, cells were harvested from each experiment group and stained with FITC-annexin V and PI (BD Biosciences) in binding buffer for 15 minutes. Stained cells were immediately subjected to flow cytometry analyses.

*Quantitative real time PCR (qRT-PCR) analysis for survivin expression*

Cells from each treatment and control groups were harvested and processed for total RNA extraction using Trizol reagent for total RNA extraction. RNA quality was checked by agarose gel electrophoresis and quantified using NanoDrop technology (Washington, DE, USA; SA Guru.,et al 2017). Complimentary DNA (cDNA) synthesis was performed by reverse transcribing 100ng/µL RNA using cDNA kit (Thermo Fisher Scientific, USA) according to the instructions provided. Briefly, RNA was mixed with the cDNA synthesis reagents to a final concentration of 100ng/µL in a final reaction mixture of 20µL. A particular volume of RNA was mixed with 2 µL of random hexamer and the volume was made up to 12 µL. This 12 µL reaction volume was incubated at 72^o^C for 5 minutes in a thermocycler. After the incubation step, 8 µL cDNA synthesis cocktail containing 4 µL cDNA buffer, 2 µL dNTPs, 1 µL reverse transcriptase enhancer and 1 µL reverse transcriptase was mixed with 12 µL RNA premix. The final reaction mixture (20 µL) was incubated at 42^o^C for 1hr and at 95^o^C for 5 minutes. The cDNA was kept at -80^o^C until used. The quantitative real time PCR (qRT-PCR) was carried out on Rotor-Gene Instrument (QIAGEN; Skelton House, Lloyd, Manchester,UK) and consisted of same steps as mentioned in our published study (Baginska et al., 2013) for evaluating expression levels of suvivin gene. The β-actin gene was used as reference housekeeping for normalisation. The primer sequences used were: Survivin F-5′CAGATTTGAATCGCGGGACCC3′ and R-5′ CCAAGTCTGGCTCGTTCTCAG 3′ for β-actin-F (5′-CGACAACGGCTCCGGCATGTGC-3′) and β-actin-R (5′-GTCACCGGAGTCCATCACGATGC-3′). Briefly, the temperature profile consisted of three segments: the first segment of the amplification cycle consisted of a denaturation program of 95^o^C for 10 minutes. The second segment consisted of three steps: denaturation (94^o^C for 15 seconds), primer annealing (58^o^C for 45 seconds), and elongation (72^o^C for 45 seconds) which was repeated for 40 cycles. The final segment consisted of a melting curve program (ranging from 35°C to 95°C).

*Statistical analysis*

Statistical analysis performed using the SPSS 16.0 software package. Chi-square analysis and Fisher exact test. The p value was considered to be significant when <0.05.

## Results

*Ex vivo Progenitor Cell Expansion and NK Cell Differentiation*

**Figure 1:**
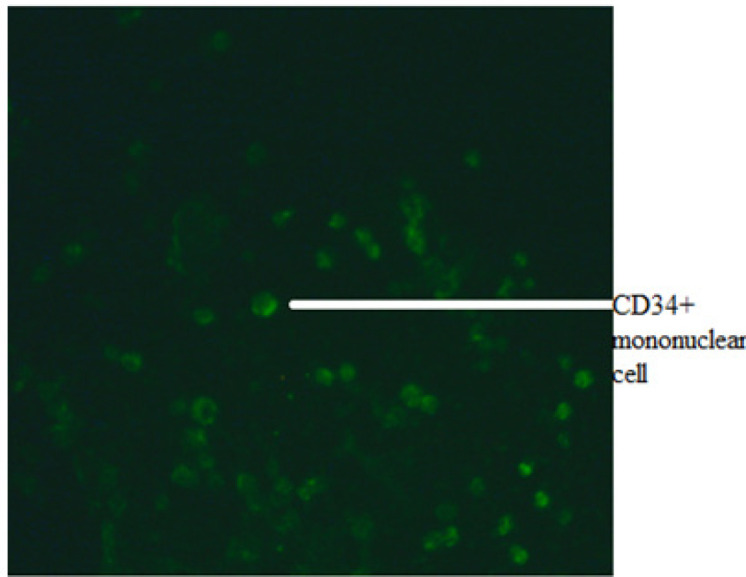
Immunofluorescence Picture of CD34+ Positive Mononuclear Cells

**Figure 2:**
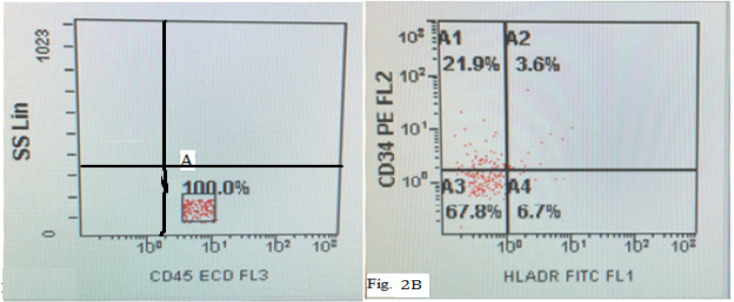
An Initial Gate (A) is set on a CD45-ECD vs SSC dot plot ,so as to contain all CD45dim and CD45 bright .This exclude CD45 negative events(i.e,red cells, platelets and other debris) Fig 2B :CD34+-PE vs HLADR-FITC dot plot and event in gate A1 shows 21.9% of positive CD34+ cells Gate A4 shows 6.7% of HLADR positive cells white gate A2 displayed 3.6% of both CD34+ and HLADR positive cells

**Figure 3:**
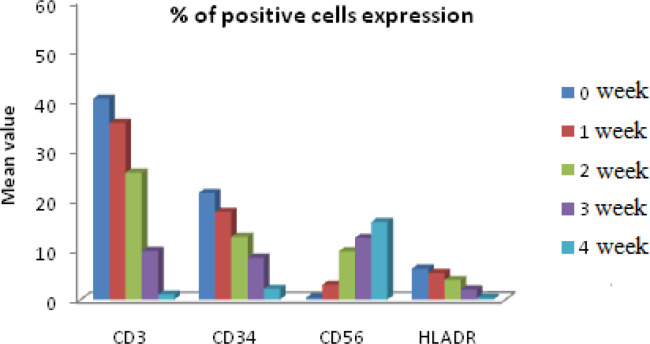
In vitro Generation of CD56 Positive Cells from Cytokine Expanded CD34+ Postive UCB Cells

**Figure 4:**
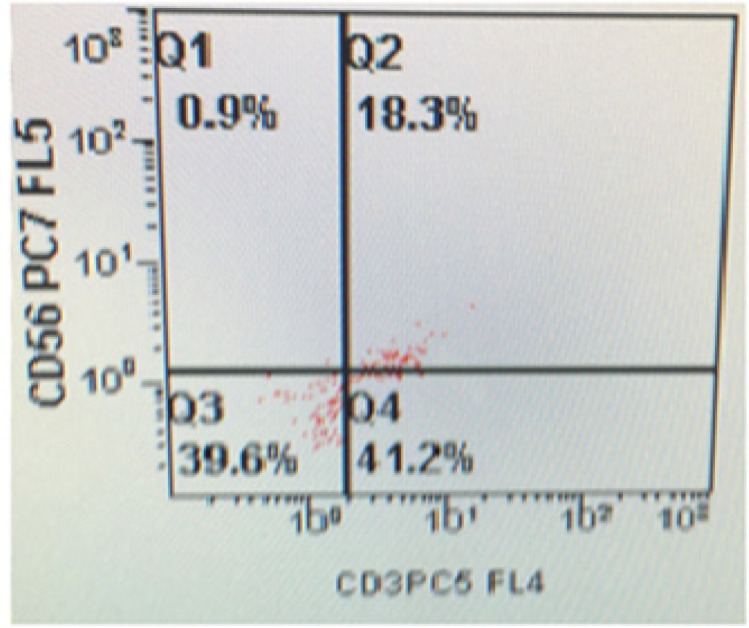
The Event in Gate Q1 Shows 0.9% of CD56 Positive NK Cells while Gate Q4 Shows 41.2% of Positive CD3 Expression during 0 Week Culture

**Figure 5:**
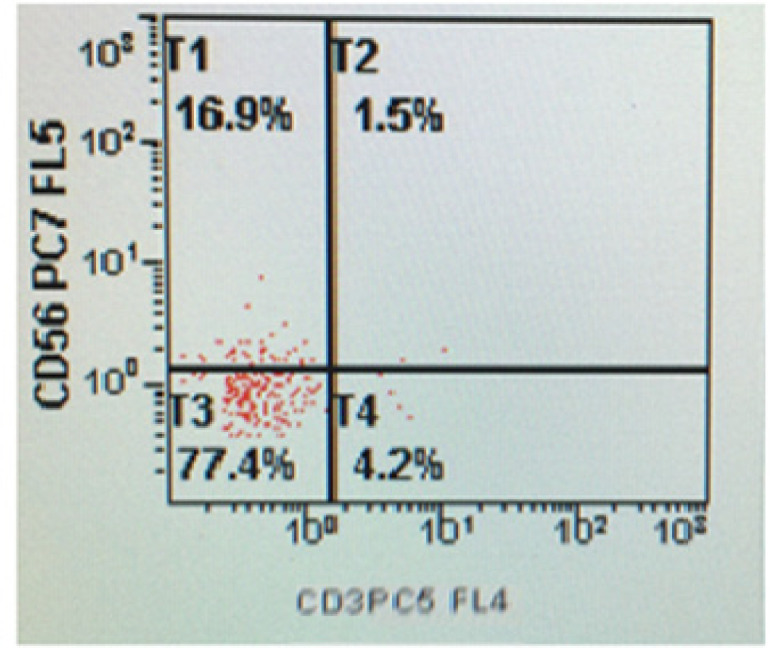
The Event in Gate T1 Shows 16.9% of CD56 Positive NK Cells after 4 Week of Cell Culture of UCB Mononuclear Cells

**Figure 6:**
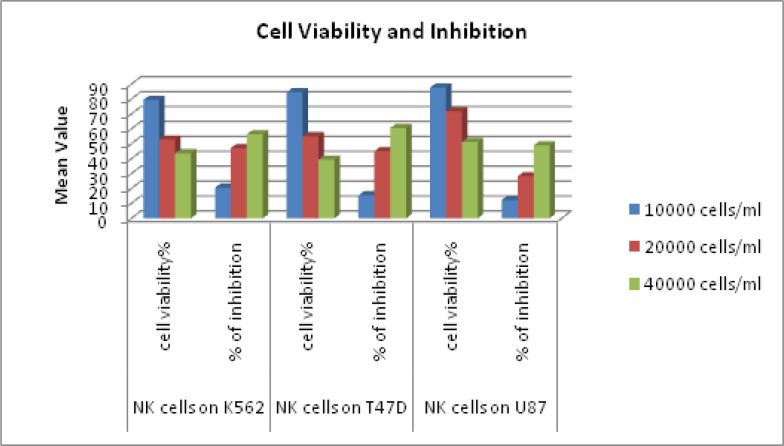
Show Percentage of Cell Viability and Inhibition of NK Cells Treated Different Tumor Cell Lines .Antitumor activity of NK cell against T47D,NK cells against U87. A: Four hour cytotoxicity against K562 cell lines with NK cells .Mean of four measurements at each E:T ration B : Four hour cytotoxicity OF NKAES-NK cells from 4 donors against AML cells from 2 patients. C: Cytotoxicity against AML cells from 5 patients after 5D of culture on MSC. Mean +SD cells killing at the indicated E:T ratios in triplicate cultures Mean +SD cells killing at the indicated E:T ratios in triplicate cultures

After the isolation of mononuclear cells from fresh obtained cord blood, we first determined the CD 34+ surface marker MNCs using immunoflorescence (Figure 1). Flowcytometric analysis revealed the CD34+ mononuclear cells during 0 week of culture as depicted in Figure (Figures 2A , 2B). This was followed by culturing these UCB-CD34+ cells with Stemline IITM stem cell expansion medium for four weeks. The CD34+ UCB cells were expanded and differentiated into NK cells which followed by log scale generation of CD56+CD3- NK cells. The differentiation step was monitored weekly and analysed for cell numbers and cell surface markers CD34, HLA-DR, CD56 and CD3 using FCM. Data were depicted as mean ± SD. The mean total cell expansion for all experiments (n=.) was 21.4± 0.5, 17.6±1, 12.6±0.8,.and fold after 0,1,2,3 and 4 weeks respectively (Figure 3). We next differentiated CD34+ UCB cells into CD56+ CD3- NK cells. First differentiation process was continued in the same expansion medium as used for CD 34+ cell expansion process followed by addition of NK cell differentiation medium which consisted of same basal medium as used for expansion supplemented with 10% FBS, the low dose cytokine cocktail as previously mentioned and a new high dose cytokine cocktail which consisted of 20 ng/mL IL-7, 20 ng/mL SCF, 1,000 U/mL IL-2 and 20 ng/mL IL-15. The medium was refreshed twice a week from day 10 onwards and plates were maintained at 37^o^C in an atmosphere containing 5% CO_2_ for 18 days. Cell number, expression of cell surface markers and purity was determined by FCM as depicted in Figures 4 and 5. The mean fold expansion of total cells after initial seeding of 1x 10^5^ cord blood mononuclear cells/ml was determined during 4 week of culture and the results are represented as mean ± SD of 4 different experiments as shown in Table 1 and also in Figure 6).

*Cytotoxicity of expanded NK cells against different tumour cells*

The cytotoxicity of NK cells expanded and differentiated from UCB was tested against different tumour cell lines such as K562 (AML cell line), U87 (Glioblastoma cell line), T47D (Breast cancer cell line). Two hundred microliters of an exponentially growing tumor cell suspension was seeded in a 96-well plate and NK cell were added at various concentrations (1x10^4^, 2x10^4^ and 4x10^4^ cells/ml). Each experiment was carried out thrice in pentaplicate for each concentration. We found that NK cells were consistently cytotoxic against all the target tumour cells with maximum cytotoxicity obtained against U87 glioblastoma cell line (Figure 6). Results are expressed as mean±SD.

*Annexin V Apoptosis Detection Using Untreated and NK cell-Treated tumour Cells*

**Figure 7:**
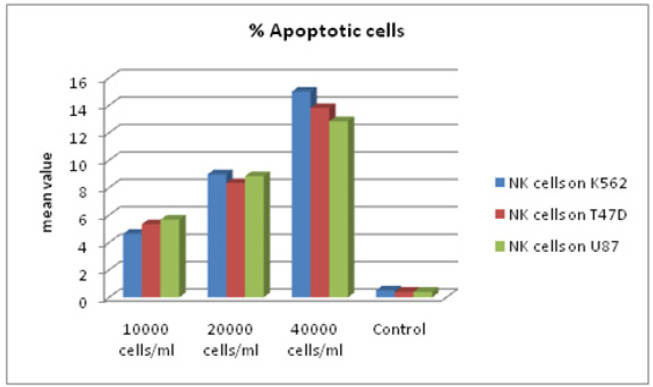
A. Flow Cytometric Analysis of Apoptosis of Different Tumor Cells Treated with NK Cells. The cells were stained with Annexin V-FITC and PI .The event in gate A1 show % of necrotic cells stained with PI and gate A3 shows % of viable cells. While gate A2 and A4 exhibit % of late apoptosis (0.7%) and early apoptosis (18.1%) respectively stained with Annexin V

**Figure 8:**
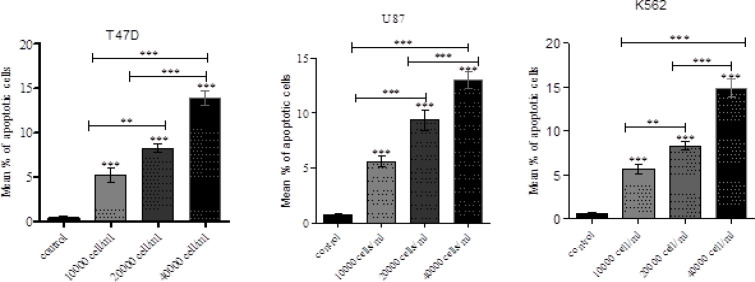
A. Mean % of Apoptosis in Different Tumor Cells .Mean % of apoptosis increased significantly in all treatment doses. Flow cytometric analysis of apoptosis of different tumor cells treated with NK cells .8B: Mean % of apoptosis in different in different tumor cells. Mean % of apoptosis increased significantly in all treatments doses

Apoptosis of target tumour cells induced by NK cells was analysed. Untreated and NK cell treated K562, U87 and T47D cells were stained using Annexin V FITC apoptosis detection kit. Data from the untreated and treated samples were obtained using Annexin V FITC assay. Figure 7 plots were used for gating cells and to identify any changes in the scatter properties of the cells. Annexin V FITC vs Propidium Iodide plots from the gated cells exhibit the populations corresponding to viable and non-apoptotic (V-PI-), early apoptosis (Annexin V+ PI-) and late apoptotic cells (Annexin V+ PI+).In the untreated (control) samples, the majority of cells (>95%) were viable and non-apoptotic (Annexin V- PI-). In contrast to cells that were treated with NK cells with different effector: target ratios for cell lines. (Time duration of treatment) we found corresponding and significant decrease in mean percentage of viable cells (Annexin V- PI-) with increase in effector NK cells in all the target tumour cells. There was an increase in early apoptotic cell populations (Annexin V+ PI-) from untreated to treated cells. A slight increase in the Annexin V+ PI+ cell population was also identified, indicating late apoptosis or dead cells. The increase in apoptotic cells was also reflected by changes in the pattern of light scatter properties for the untreated and treated groups. During apoptosis, cell shrinkage is associated with decrease in forward scatter. Moreover, the formation of apoptotic vesicles in the cells during apoptosis leads to an increased side scatter profile as depicted in Tables 2A, 2B, 3C and also presented in Figures 7 and 8A and 8B). 

*NK cell treatment and Survivin expression in tumour cell lines*

**Figure 9:**
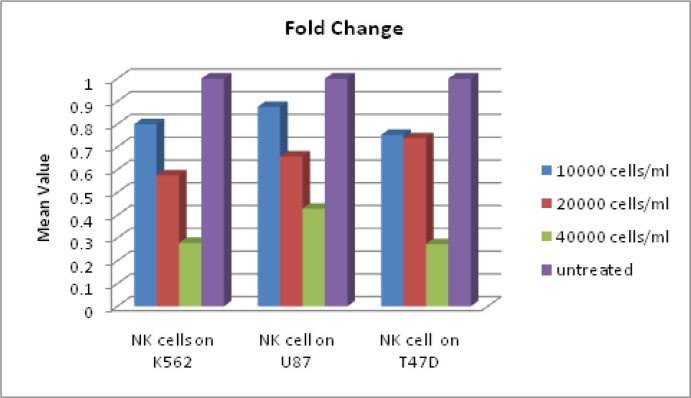
Shows Fold Change of Survivin Expression of NK Cells Treated Tumor Cells

**Figure 10:**
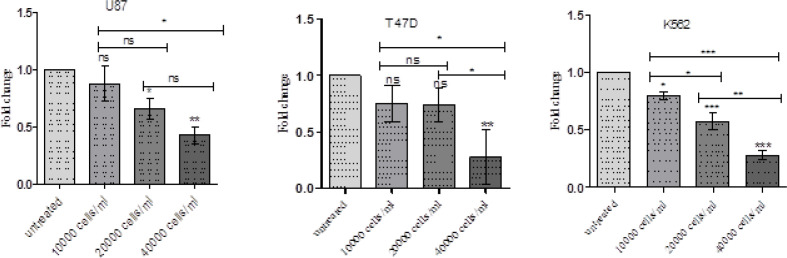
NK Cells Significantly Reduce Survivin Gene Expression in Different Cancer Cell Lines in vitro and the Reduction was Observed to be more on Increasing NK Cell Proportion

We analysed the effect of NK cell treatment on survivin gene expression in different tumour cells K562, U87 and T47D in vtro. We observed that survivin gene expression decreased in tumor cells on treatment with NK cells. Further, it was identified that there was a progressive and statistically significant decrease in survivin gene expression with increasing NK cell dose. The results are expressed as mean±SD and represented in Tables 3A, 3B, 3C and Figures 9 and 10.

**Table 1.** Fold Expansion of Total Cells During 4 Week of Culture

Group	Mean fold expansion ±SD (n=4)	95% confidence interval
1 week	36.750±8.421	23.352 - 50.148
2 week	108.00±9.092	93.535 - 122.47
3 week	612.50±158.82	359.82 - 865.18
4 week	1925.0±434.93	1233.0 - 2617.0

**Table 2:** Mean Percentage of Apoptotic Cells in NK Cells Treated Tumour Cells at Different NK Cell Concentrations

		Mean		Std. Deviation			Std. Error		95% Confidence Interval for Mean				
									Lower Bound		Upper Bound		
10,000 cells/ml		5.3333		1.26623			0.73106		2.1878		8.4788		
20,000 cells/ml		8.3333		0.70946			0.40961		6.5709		10.0957		
40,000 cells/ml		13.8		0.6245			0.36056		12.2487		15.3513		
Control		0.4		0.2			0.11547		-0.0968		0.8968		
10,000 cells/ml	5.6667				1.30512	0.75351		2.4246				8.9088		
20,000 cells/ml	8.8333				0.41633	0.24037		7.7991				9.8676		
40,000 cells/ml	12.8333				1.2897	0.74461		9.6295				16.0371		
Control	0.4				0.36056	0.20817		-0.4957				1.2957		
10,000 cells/ml			4.6333	1.16762		0.67412				1.7328			7.5339		
20,000 cells/ml			8.9667	1.51438		0.87433				5.2047			12.7286		
40,000 cells/ml			15	0.26458		0.15275				14.3428			15.6572		
Control			0.5	0.36056		0.20817				-0.3957			1.3957		

**Table 3:** Mean Expression of Survivin m-RNA after NK Cell Treatment

	Mean	Std. Deviation	Std. Error	95% Confidence Interval for Mean	
				Lower Bound	Upper Bound
10,000 cells/ml	0.7528	0.16314	0.09419	0.3475	1.158
20,000 cells/ml	0.7378	0.14967	0.08641	0.366	1.1096
40,000 cells/ml	0.2737	0.24355	0.14061	-0.3313	0.8788
untreated	1	0	0	1	1
	Mean	Std. Deviation	Std. Error	95% Confidence Interval for Mean	
				Lower Bound	Upper Bound
10,000 cells/ml	0.8758	0.15142	0.08742	0.4996	1.2519
20,000 cells/ml	0.6579	0.16215	0.09362	0.2551	1.0607
40,000 cells/ml	0.4289	0.12873	0.07432	0.1091	0.7487
untreated	1	0	0	1	1
	Mean	Std. Deviation	Std. Error	95% Confidence Interval for Mean	
				Lower Bound	Upper Bound
10,000 cells/ml	0.7998	0.05708	0.03296	0.658	0.9416
20,000 cells/ml	0.575	0.12502	0.07218	0.2645	0.8856
40,000 cells/ml	0.2785	0.06745	0.03894	0.1109	0.4461
untreated	1	0	0	1	1

## Discussion

Cancer immunosurveillance is a protective mechanism of the body against cancer and a major role is played by natural killer cells. NK cells are the first line defence mechanism against malignant transformed and virally infected cells (Conlon et al., 2015).However, malignant cells evolve a mechanism to evade the defence mechanisms of immune system and create an immunosuppressive microenvironment (Almand et al., 2001; Deng et al., 2013). This is brought out by secreting some factors such as MICA, MICB and vesicles containing ULBP3, by tumour cells which impair cytotoxic ability of NK cells thus facilitating immune evasion (Ib et al., 2011; Fernández-Messina et al., 2010).

In addition, NK cells also secrete tumour cell-derived factors and tumour derived exosomes which further suppress NK cell activity (Baginska et al., 2013). All of these factors cause decreased expression of activating receptors on NK cells while increasing expression of the receptors having an inhibitory role (Ib et al., 2011; Fernández-Messina et al., 2010). Several studies have identified that invitro expanded NK cells have an increased cytotoxic efficiency and can be used for cancer treatment (Mamessier et al., 2011; Ascierto et al., 2013). It has been identified that expanded NK cells from healthy donors possess higher cytotoxic potentials than NK cells isolated from cancer patients (Denman et al., 2012; Lim et al., 2013). The aim of this study was to explore the cytolytic activities of cord blood derived CD34+ differentiated and invitro expanded NK cells. The cytolytic activities of these expanded NK cells were tested against K562 leukemic cell lines, T47D breast cancer cell lines and U87 glioma cell lines. 

Our results indicate that expanded NK cells exhibit almost similar cytolytic effects on all the three types of cells used in this study although a slightly higher level of cytolytic activity was observed against K562 leukemic cell lines. Previous studies report that expanded NK cells show variable cytotoxicity against different cancer cell lines (Berg et al., 2009). The expanded NK cells have been reported to attack primary patient cells isolated from acute myeloid leukaemia and chronic myeloid leukaemia patients (Tanaka et al., 2012; Mir et al., 2017). CML is a hematopoietic stem-cell disorder and is induced by the BCR-ABL oncogene, whose gene product is a BCR-ABL tyrosine kinase . The inability of BCR-ABL kinase inhibitors to completely kill leukemia stem cells (LSCs) indicates that these kinase inhibitors are unlikely to cure CML (Mir et al., 2015, Mir et al., 2015).

The use of invitro expanded NK cells is gaining importance as cancer immune therapeutic strategy.

Allogeneic NK cells have been observed to have strong anticancer effects after haploidentical hematopoietic stem cell transplantation (haplo-HCT) in patients with advanced AML (Ruggeri et al., 2002). Haplo-HCT has been suggested as a viable treatment option for AML patients lacking a matched sibling donor and peripheral blood is being suggested to be considered as graft source for haplo-HCT with acceptable post-transplant outcomes (38). 

The mechanisms employed for evading immune surveillance by cancer cells include among others down regulation of surface expression of NKG2D ligands ULBP1, ULPB2 and MICA. These ligands help NK cells to recognize tumour cells and render them susceptible to cytolysis (Rashidi et al., 2016). A large number of studies are being performed presently to unravel the innate immune mechanisms for target cell recognition on one hand and the multifaceted lytic machinery of NK cells on the other hand. The ability of manipulating not only the balance of receptors involving activating and inhibitory signals in NK cells but also their cognate ligands and the sensitivity of tumour cells to apoptosis will lead to new perspectives in NK cell based immunotherapy.

In conclusion, our finding indicated that NK cells differentiated from CD34+ cells isolated from cord blood were able to induce apoptosis and has shown increased antitumor potential in vitro against different cancer cells besides cause down regulation of survivin gene expression in tumor cells. Therefore, NK cell therapy represents a promising immunotherapy for cancers like AML and other haematological malignancies. Furthers studies are necessary to confirm our findings. 
